# Irradiance Sensing through PV Devices: A Sensitivity Analysis

**DOI:** 10.3390/s21134264

**Published:** 2021-06-22

**Authors:** Antonino Laudani, Gabriele Maria Lozito, Francesco Riganti Fulginei

**Affiliations:** Dipartimento di Ingegneria, Università degli Studi Roma Tre, Via Vito Volterra 62b, 00146 Roma, Italy; gabrielemaria.lozito@uniroma3.it (G.M.L.); riganti@uniroma3.it (F.R.F.)

**Keywords:** photovoltaics, solar energy, solar irradiance, sensitivity analysis, monitoring

## Abstract

In this work, a sensitivity analysis for the closed-form approach of irradiance sensing through photovoltaic devices is proposed. A lean expression to calculate irradiance on a photovoltaic device, given its operating point, temperature and equivalent circuit model, is proposed. On this expression, the sensitivity towards errors in the measurement of the photovoltaic device operating point and temperature is analyzed, determining optimal conditions to minimize sensitivity. The approach is studied for two scenarios, a stand-alone sensor and irradiance sensing on an operating power-producing photovoltaic device. A low-cost realization of a virtual sensor employing the closed form for monitoring performance of photovoltaic module is also presented, showing the advantage of this kind of simple solution. The proposed solution can be used to create a wireless sensor network for remote monitoring of a photovoltaic plant, assessing both electrical and environmental conditions of the devices in real time.

## 1. Introduction

Irradiance knowledge is a critical asset in the production of energy from photovoltaic (PV) conversion and its measurement, estimation and forecasting are a very active area of research [1]. This quantity expresses the power density of solar radiation that reaches the photovoltaic device. Irradiance directly influences the electrical characteristic of the PV device [2,3], resulting in a different operating point and maximum deliverable power [4]. In a similar fashion, temperature also affects the electric characteristic of the PV device. However, temperature can be easily measured by a back-side sensor, or through some functional relation linking the PV device temperature to the ambient temperature [5]. Irradiance, on the other hand, is strongly unpredictable due to the weather conditions, can change rapidly and is difficult to estimate.

Irradiance measurement is often an appropriate solution to monitor plant electrical behaviour, optimally reconfigure the panels connections to avoid partial shading [6] and aiding MPPT (maximum power point tracking) systems to ensure maximum power is delivered from the device [7]. Irradiance measurement is often coupled with forecasting techniques [8,9,10] for short and long term predictions, which is especially useful when the management of storage systems is involved [11]. Moreover, fault and aging detection [12] are two applications that can greatly benefit from an on-site implementation. Unfortunately, the irradiance estimation is usually performed by means of very expensive devices, such as pyranometers; this kind of device can be quite accurate in the evaluation of solar radiation, but, at the same time, it does not take into account the energy generation process inside the PV module (highly dependent on the technology and absorption characteristic of the materials used). This makes the pyranometer approach more related to solar thermal plants than PV ones. A suitable alternative is to use small PV reference cells, which are appositely built for sensing irradiance application (these devices are usually addressed as solarimeters); the use of this devices can give a more effective evaluation of irradiance components involved in PV energy generation, especially if the cell is built using the same material used for the PV system. A further advantage of this solution is the possibility to tilt the reference cell rigidly with the PV modules, taking into account this effect as well. The cost of such PV reference cell is not prohibitive, but, in many cases, is comparable to that of a PV module. A low-cost alternative is to use a normal PV cell, coupled with a temperature sensor, and build a virtual sensor by exploiting artificial intelligence and implement it in a microcontroller, or a FPGA [13].

The simplest solution which allows an accurate way of measuring irradiance is through measurements on the PV device itself. The approach assumes that the device electrical behaviour is represented accurately by the single-diode model. Under this assumption, a functional relationship binds voltage, current, temperature and irradiance. This functional relationship can be either fitted through numerical methods [13], or analytically expressed through manipulation of the current–voltage relationship of the circuit model [14]. This methodology was validated experimentally and was proved accurate and reliable. Still, the functional relationship defined is non-linear and the measurements can be subject to errors and uncertainties.

In this work, a sensitivity analysis with respect to the measurements is performed on the relationship derived in [14]. The purpose of this work is to identify the optimal setups to achieve a robust measurement for which the perturbation induced by errors is minimized. Two setups are considered. The first one involves the irradiance measurement on a PV device that is actually producing power. Since the PV device is operative, its operating point cannot be changed (and will most likely lie near the maximum point); this means that current, voltage and temperature will be measured separately. The second one involves the irradiance measurement on a PV device that is dedicated only to this particular function (i.e., a solarimeter). In this case, the operating point can be set by using a fixed resistive load and only two measurements (voltage and temperature) are required. The two approaches are similar and are fundamentally based on the same equation, but exhibit very different sensitivities, as will be shown.

In addition, a low-cost prototype for a monitoring system, exploiting the irradiance close formula, was implemented using the microcontroller ESP32 by EXPRESSIF. An experimental test involving a PV emulator was carried out to validate the approach.

The proposed approach, together with the prototype implementation, aims at creating a sustainable and scalable solution for the environmental and electrical monitoring of PV power plants [15]. Assessing direct irradiance from meteorological data, or by regressing time series, is indeed possible, but considerably imprecise, if compared to direct estimation. Assuming this assessment is used for forecasting, the error propagation can lead to very poor predictions. Considering that estimated and forecast irradiance are the most influential quantities to assess the produced energy in a PV power plant (and can be used in conjunction with fault assessment methodologies [16,17]), the solution proposed in this work can have a large impact in many optimization strategies for renewable energy communities and micro-grids [18,19,20].

The paper is structured as follows. First, a brief derivation of the irradiance expression will be given, starting from the single-diode model circuit characteristic. Then, the sensitivity analysis for the proposed function will be discussed, with special reference to the two different approaches. Following, individual and total sensitivity analysis results will be presented with special focus on the optimal operating points to minimize the perturbation effects. Lastly, the prototype implementation for the monitoring system and the experimental validation will be presented. Conclusions and final remarks will conclude the paper, with a short Appendix A summarizing the quantities and unity of measurements used.

## 2. Materials and Methods

In this section, the full analysis leading to the sensitivity of the measured irradiance on a PV device will be presented. First, a brief summary of the results presented in [14] will be discussed, where the closed form of the irradiance for a PV device is derived. Then, the sensitivity analysis of this function with respect to its main variables will be proposed. Lastly, the modelling part relative to two real scenarios will be determined, the first relative to the measurement of irradiance on an operative, power-producing PV device and the second relative to a stand-alone sensor.

### 2.1. Closed-Form Expression for Irradiance on a PV Device

**Figure 1:**
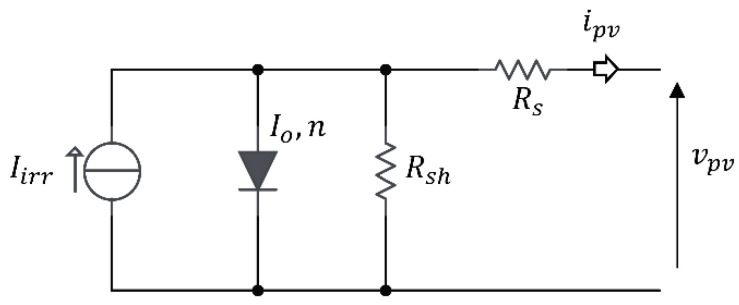
Single diode circuit model for a PV device.

Irradiance sensing on a PV device is possible, assuming that the electric behavior of the device is represented by its equivalent five-parameter, “single diode” model. The model is represented in Figure 1. The current–voltage relationship is given in Equation (1):

(1) $$i_{pv}=I_{irr}-I_{o}\left(exp\left(\frac{v_{pv}+R_{s}i_{pv}}{nV_{T}}\right)-1\right)-\frac{v_{pv}+R_{s}i_{pv}}{R_{sh}}$$

where $R_{s}$ is the series resistance, $R_{sh}$ is the shunt resistance, $I_{irr}$ is the irradiance current, $I_{o}$ is the reverse saturation current of the diode, n is the modified ideality factor of the diode (scaled by the number of cells in series) and $V_{T}$ is the thermal voltage. The circuit parameters are unique to a PV device and change their value according to the environmental conditions of device temperature T and incident solar irradiance G. Their numerical values can be identified at standard reference conditions (SRC), with a temperature of $T_{ref}=298.15\;K$ and $G_{ref}=1000\;W/m^{2}$, by exploiting datasheet information.

The subscript *ref* indicates a parameter identified at SRC. Assuming parameters at SRC are known, their values for arbitrary irradiance and temperatures can be computed by means of the following equations [3]:

(2) $$R_{s}=R_{s,ref}$$

(3) $$R_{sh}=R_{sh,ref}\cdot \frac{G_{ref}}{G}$$

(4) $$I_{irr}=I_{irr,ref}\cdot \frac{G}{G_{ref}}\cdot \left(1+\alpha_{T}\left(T-T_{ref}\right)\right)$$

(5) $$I_{o}=I_{o,ref}\cdot {\left(\frac{T}{T_{ref}}\right)}^{3}e^{\frac{E_{g,ref}}{k\cdot T_{ref}}-\frac{E_{g}\left(T\right)}{k\cdot T}}$$

(6) $$n=n_{ref}$$

where $\alpha_{T}$ is the temperature coefficient for the short circuit current of the device, $E_{g}\left(T\right)$ is the bandgap energy of silicon in eV expressed as function of *T* (note that $E_{g,ref}=E_{g}\left(T_{ref}\right)$) and *k* is the Boltzmann constant in eV/K.

By injecting Equations (2)–(5) in Equation (1) and re-arranging some terms, it is possible to express all parameters dependence by temperature and irradiance:

(7) $$\begin{array}{ll} \frac{G}{G_{ref}}(I_{irr,ref}^{\;}+ & \alpha_{T}\left(T-T_{ref}\right)-\frac{v_{pv}+i_{pv}R_{s,ref}^{\;}}{R_{sh,ref}^{\;}})\\ & =i_{pv}+I_{o,ref}{\left[\frac{T}{T_{ref}}\right]}^{3}\exp\left[\frac{E_{g,ref}}{kT_{ref}}-\frac{E_{g}}{kT}\right]\left[\exp\left(\frac{v_{pv}+i_{pv}R_{s,ref}^{\;}}{nV_{T}}\right)-1\right] \end{array}$$

Lastly, through some simple manipulations:

(8) $$G=G_{ref}\frac{i_{pv}+I_{o,ref}{\left[\frac{T}{T_{ref}}\right]}^{3}\exp\left[\frac{E_{g,ref}}{kT_{ref}}-\frac{E_{g}}{kT}\right]\left[\exp\left(\frac{v_{pv}+i_{pv}R_{s,ref}^{\;}}{nV_{T}}\right)-1\right]}{\left(I_{irr,ref}^{\;}+\alpha_{T}\left(T-T_{ref}\right)-\frac{v_{pv}+i_{pv}R_{s,ref}^{\;}}{R_{sh,ref}^{\;}}\right)}$$

**Figure 2:**
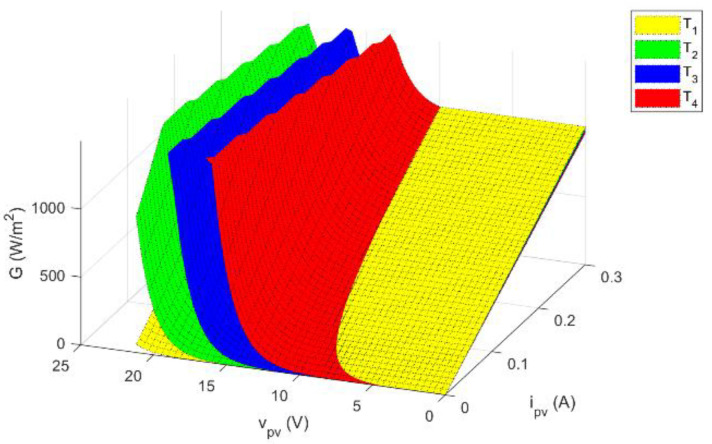
Irradiance as a function of voltage and current for different temperatures, where $T_{4}>T_{3}>T_{2}>T_{1}$.

As can be seen, given the knowledge of the single-diode model parameters at SRC, the irradiance can be calculated as a function of the operating point of the device ($v_{pv},\;i_{pv})$ and temperature T. As can be seen from Figure 2, the behavior of Equation (8) is of a smooth and continuous function.

### 2.2. Sensitivity Analysis

Through Equation (8), it is possible to use a photovoltaic device as a virtual measurement instrument for solar irradiance, involving the measurements of voltage, current and temperature of the PV Cell. Clearly, the basis of this formula is the assumption that Equations (2)–(6) are accurate enough in describing the parameters dependence from G and T. These can be considered valid in the typical range of PV application, as it is demonstrated by their large use in the solar simulators (such as the SAM model [21]). Nevertheless, two further main sources of error should be considered in a practical application. The first one is linked to the identification of the five parameters of the single diode model. These parameters can be extracted starting from datasheet information at SRC or by the measurement of a single current–voltage curve in some controlled conditions of temperature and irradiance for the specific device used. In the first case, there are different opinions about the best way to proceed. The authors suggest the reading of the paper [22], which gives insight into the problem of the identification and also proposes an analytical way to evaluate the boundary for all these parameters. Clearly, datasheet information reports average quantities for the so-called primary parameters (short circuit current $I_{SC}$, open circuit voltage $V_{OC}$ and maximum power point current $I_{MP}$ and voltage $V_{MP}$). This may result in erroneous identified parameters.

A strategy to robustly identify the five-parameter model was proposed in [23]. On the other hand, the process of the measurement of the current–voltage curve and identification of the five-parameter model is surely a more effective strategy (for example, employing reduced forms or the TSLLS method [24]), even if requires tailored equipment for measurements, leading to a more complicated procedure. Assuming the model is accurately identified, the second source of errors is the measurement of voltage, current and temperature, whose noise can lead to inaccurate estimations of irradiance. It is necessary to evaluate the weight of these error sources to obtain a robust setup. In addition, these two sources of errors have a different behavior, since the former (the one related to the five parameters or to the model) can be seen as bias error, while the latter (related to the measurements of the voltage, current and temperature) is linked to the measurement accuracy of these quantities. The aim of the following sensitivity analysis is to verify the weights of these error sources and propose solutions allowing a reduction in their effects. Assuming a quantity is related to one or more variables through a functional relationship, such as irradiance is related to voltage, current and temperature through Equation (8), the sensitivity of that quantity with respect to the variables can be expressed as:

(9) $$S_{X_{i}}^{G}\left(X\right)=\frac{\partial G}{\partial X_{i}}\cdot \frac{X_{i}}{G}$$

where $X=\left\{v_{pv},i_{pv},T\right\}$ and $X_{i}$ is one of the three variables related to the irradiance. Sensitivity can be used to quantify the error propagation from the variables to the quantity. Indeed, the error in G evaluation, due to error in the measurement of voltage $v_{pv}$, current $i_{pv}$ and temperature T, is given by:

(10) $$\frac{\Delta G}{G}=S_{v_{pv}}^{G}\frac{\Delta v_{pv}}{v_{pv}}+S_{i_{pv}}^{G}\frac{\Delta i_{pv}}{i_{pv}}+S_{T}^{G}\frac{\Delta T}{T}$$

where the terms $\Delta i_{pv}/i_{pv}$, $\Delta v_{pv}/v_{pv}$ and ΔT/T are just the previously stated relative errors. As can be seen, sensitivity itself is a function of the variables *X*. This can be used as an advantage in design, if a minimum of Equation (9) can be found, by designing a system that operates in the proximity of a point with a minimum sensitivity, resulting in a more robust measurement. Although Equation (8) is an analytical form and the expressions of the sensitivity by Equation (9), with respect to different parameters, can be computed analytically, they would not give any insight for design consideration. For example, the analytic expression for the $S_{V}^{G}$ (which is the simplest to compute), assuming that $N\left(v_{pv},i_{pv},T\right)$ is the numerator of Equation (8) and $D\left(v_{pv},i_{pv},T\right)$ is the denominator, assumes the following form:

(11) $$\begin{array}{ll} S_{v_{pv}}^{G}=S_{v_{pv}}^{N}-S_{v_{pv}}^{D} & =\frac{\partial N}{\partial v_{pv}}\cdot \frac{v_{pv}}{N}-\frac{\partial D}{\partial v_{pv}}\cdot \frac{v_{pv}}{D}\\ & =\frac{G_{ref}I_{0,ref}}{nV_{T}}{\left[\frac{T}{T_{ref}}\right]}^{3}\exp\left[\frac{E_{g,ref}}{kT_{ref}}-\frac{E_{g}}{kT}\right]\left[\exp\left(\frac{v_{pv}+i_{pv}R_{S,ref}^{\;}}{nV_{T}}\right)\right]\cdot \frac{v_{pv}}{N}\\ & +\frac{1}{R_{SH,ref}^{\;}}\cdot \frac{v_{pv}}{D}\\ & =\{\frac{\frac{I_{0,ref}}{nV_{T}}{\left[\frac{T}{T_{ref}}\right]}^{3}\exp\left[\frac{E_{g,ref}}{kT_{ref}}-\frac{E_{g}}{kT}\right]\left[\exp\left(\frac{v_{pv}+i_{pv}R_{S,ref}^{\;}}{nV_{T}}\right)\right]}{i_{pv}+I_{0,ref}{\left[\frac{T}{T_{ref}}\right]}^{3}\exp\left[\frac{E_{g,ref}}{kT_{ref}}-\frac{E_{g}}{kT}\right]\left[\exp\left(\frac{v_{pv}+i_{pv}R_{S,ref}^{\;}}{nV_{T}}\right)-1\right]}\\ & +\frac{1}{\left(R_{SH,ref}^{\;}I_{irr,ref}^{\;}+R_{SH,ref}^{\;}\alpha_{T}\left(T-T_{ref}\right)-v_{pv}+i_{pv}R_{S,ref}^{\;}\right)}\}\cdot v_{pv} \end{array}$$

For this reason, in the following discussion, a numerical approach is followed in the computation of the partial derivative in Equation (9). The three sensitivities considered herein are:

(12) $$S_{v_{pv}}^{G}\left(v_{pv},i_{pv},T\right)=\frac{G\left(v_{pv}+\Delta v_{pv},i_{pv},\;T\right)-G\left(v_{pv},i_{pv},\;T\right)}{\Delta v_{pv}}\cdot \frac{v_{pv}}{G}$$

(13) $$S_{i_{pv}}^{G}\left(v_{pv},i_{pv},T\right)=\frac{G\left(v_{pv},i_{pv}+\Delta i_{pv},\;T\right)-G\left(v_{pv},i_{pv},\;T\right)}{\Delta i_{pv}}\cdot \frac{i_{pv}}{G}$$

(14) $$S_{T}^{G}\left(v_{pv},i_{pv},T\right)=\frac{G\left(v_{pv},i_{pv},\;T+\Delta T\right)-G\left(v_{pv},i_{pv},\;T\right)}{\Delta T}\cdot \frac{T}{G}$$

where the variables perturbations $\Delta \left\{v_{pv},i_{pv},\;T\right\}$ are computed as 0.01% of the perturbed variable value.

### 2.3. Two Approaches: Three-Measurement and Two-Measurement

**Figure 3:**
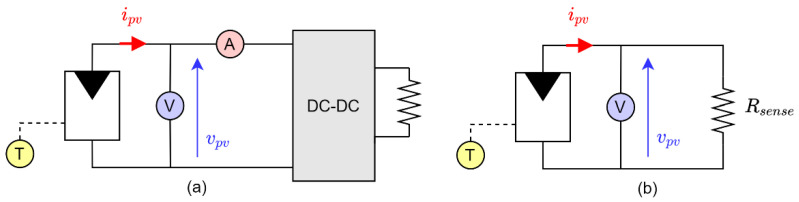
Three-measurement approach for an operative PV device (**a**) and two-measurement approach for a stand-alone sensor (**b**).

Commonly, PV devices operate to produce electric power. Due to their non-linear electric characteristic, a DC-DC converter is employed to ensure maximum power transfer to the load. Assessing irradiance through Equation (8) on an operative PV device is possible and requires three independent measurements. A classic setup can be seen in Figure 3a, where the dual measurement of current and voltage can be easily implemented using a digital power meter [25,26]. In general, those quantities are always monitored for maximum power point tracking purposes and, for this reason, it is straightforward to include them in this manner. However, smaller PV devices, such as monocrystalline cells, can be used just for the purpose of irradiance measurement. In this case, since no real load is present and due to the implicit difficulty of measuring current, a setup such as the one in Figure 3b is used, where the current is directly derived from Ohm’s law. For this second setup, the current is assumed as $i_{pv}=v_{pv}/R_{sense}$. The second setup can be implemented using as voltage sensor the ADC input of almost any microcontroller available. The drawback lies in the fact that the voltage measurement affects two variables and the second one is also theoretically affected by the measurement error of the resistance (even if this can be reduced during the setup of the sensor). The use of Ohm’s law could suggest the possibility of expressing the sensitivity, with respect to current, by means of the sensitivity towards the ratio of two quantities, which is, by definition:

(15) $$S_{i_{pv}}^{G}=S_{v_{pv}/R_{sense}}^{G}=\frac{1}{2}S_{v_{pv}}^{G}-\frac{1}{2}S_{R_{sense}}^{G}$$

Equation (15), in this case, leads to erroneous evaluation, since the link between the three parameters $v_{pv},i_{pv},\;R$ is given by Ohm’s law coupled with Equation (1). Thus, the sensitivity towards current (13) is substituted by a sensitivity towards the sensing resistance, as shown in Equation (16):

(16) $$S_{R}^{G}\left(v_{pv},v_{pv}/R,T\right)=\frac{G\left(v_{pv},v_{pv}/\left(R_{sense}+\Delta R_{sense}\right),\;T\right)-G\left(v_{pv},v_{pv}/R_{sense},\;T\right)}{\Delta R_{sense}}\cdot \frac{R_{sense}}{G}$$

It is worth noticing that even the $S_{v_{pv}}^{G}$ should be computed in a different way, since any error on $v_{pv}$ influences in a different way the new G expression:

(17) $$S_{v_{pv}}^{G}\left(v_{pv},v_{pv}/R,T\right)=\frac{G\left(v_{pv}+\Delta v_{pv},(v_{pv}+\Delta v_{pv})/R_{sense},\;T\right)-G\left(v_{pv},v_{pv}/R_{sense},\;T\right)}{\Delta v_{pv}}\cdot \frac{v_{pv}}{G}$$

## 3. Results

Numerical tests were performed to gain insight on the sensitivity, considering three PV devices, with the five-parameter model reported in Table 1, along with the datasheet parameters used for identification. The first one is relative to a 315W module STP315S-20, commonly used for power production, and is implemented in the measurement setup of Figure 3a. The second is a small mono-Si cell KXOB22-01X8L and is implemented in the measurement setup of Figure 3b. The third is a small power mono-Si cell MSP1M210-18-6W, usually used for the supply of IoT devices, such as wireless sensors or small appliances, but that could be used also for small measurement setups and, for this reason, it is herein used for the comparison of the two solutions.

The five-parameter model for these devices was identified by using the reduced forms and the procedure described in [27]. During the identification, the modified ideality factor *n* was chosen taking into account the possibility to have *I_o,ref_* current as low as possible; this is motivated by the fact that this coefficient in Equation (8) multiplies an expression highly dependent on temperature T and, consequently, this choice reduces the sensitivity of these parameters to this quantity. On the other hand, for the third device, in order to show that this is not a necessary constraint, the ideality factor was fixed equal to 1.3 × 36. In the following, four quantities are computed, $S_{v_{pv}}^{G}$, $S_{i_{pv}}^{G}$,$\;S_{T}^{G}$ and $S_{R}^{G}$, according to the numerical expressions previously stated. The results for both the configurations with three- and two-measurement approaches are shown. It is worth noticing that both cases have three sensitivities: $S_{v_{pv}}^{G}$, $S_{i_{pv}}^{G}$,$\;S_{T}^{G}$ for the three-measurement case and $S_{v_{pv}}^{G}$, $S_{R}^{G}$,$\;S_{T}^{G}$ for the two-measurement one.

In the computation of these quantities, a complete mathematical approach is theoretically possible, without considering the practical aspects of the PV application. However, this would lead to results with very low practical interest. Instead, a simpler approach, based on application-specific choices, was followed. In particular, the sensitivities are studied considering three different couples of irradiance and temperature operating points, analyzing the behavior of sensitivities in correspondence of these couples. This choice is due to the fact that the temperature of the module is strictly linked to ambient temperature, but it is also strongly influenced by the incident radiation on the module. The three operating points are reported in Table 2.

To derive an insight useful for design, the sensitivities behavior is computed in function of only one parameter among $v_{pv},i_{pv}$ and R. Indeed, these three parameters are not independent, since they must satisfy both Equation (1) and Ohm’s law. However, the most useful choice is to use $v_{pv}$ for the three-measurement case and R in the two-measurement one. The choice of R can be seen as natural, since it can be related to the existence of a best value for the sensing resistance; the choice of $v_{pv}$ is also obvious, since the voltage is used in many DC-DC converters as regulation parameter coupled with duty cycle. Showing the sensitivity with respect to the device voltage is useful to identify the operating conditions, leading to robust irradiance measurement conditions. For the third PV module, since it is herein reported for comparison purposes, the sensitivities are always plotted versus voltage $v_{pv}$.

### 3.1. Voltage Sensitivity

**Figure 4:**
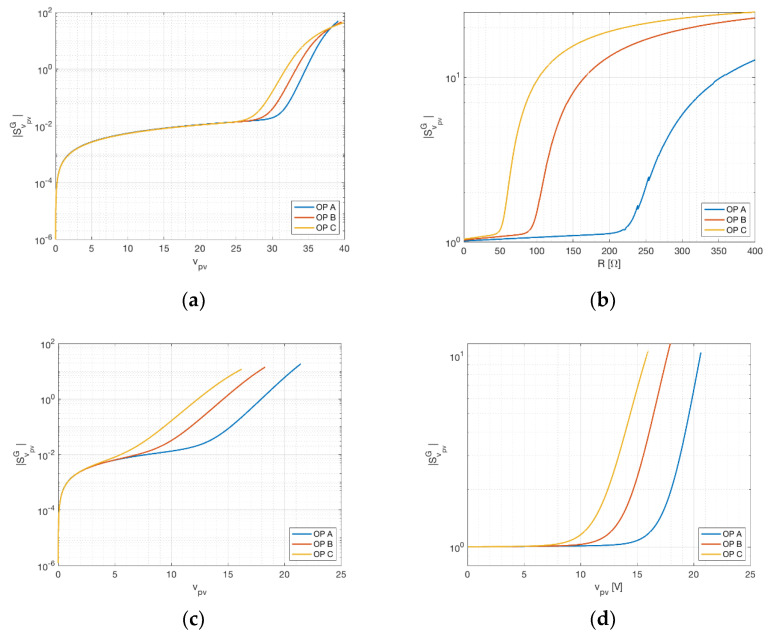
Voltage sensitivity of the irradiance at different operating points (OP). (**a**) The three-measurement approach for STP315S-20. (**b**) The two-measurement approach for KXOB22-01X8L. (**c**) The three-measurement approach and (**d**) the two-measurement one for MSP1M210-18-6W.

In this paragraph, the sensitivity of the irradiance towards perturbations on the voltage measurement is analyzed. In Figure 4, the sensitivity calculated by means of Equations (12) and (17) is reported against the PV device voltage, for the three different operation points previously stated. Figure 4a represent the three-measurement approach on the STP315S-20, Figure 4b the two-measurement approach on the KXOB22-01X8L. In the three-measurement approach, as expected, the sensitivity is lower for operating points near short circuit (i.e., $v_{pv}=0$) and rises quickly for increasing voltages. A remarkable knee is visible in the curves of Figure 4a close to MPP condition; the sensitivity assumes almost negligible values (<$10^{-2}$) before this knee, whereas it maintains values of order of 1 around the MPP. In the two-measurement approach, it is possible to repeat a similar discussion, but this time versus the sensing resistor. It is worth noticing that a small value of resistance can be seen as a load line in the v–i plane with a high slope and, consequently, an operating point close to short circuit. On the other hand, high values of resistance result in an operating point near open circuit. For this reason, the knees visible in Figure 4b for different operating points are again related to MPP conditions, which happen for different equivalent resistor values (from 50 Ω, for operating point C, to 230 Ω, for operating point A). Even in this case, the sensitivity is lower before the MPP and higher after. It is worth noticing that the sensitivity values are not negligible in the two-measurement approach and this is because in this approach the voltage weighs indirectly on the current, which, as it is shown in the next paragraph, has a higher sensitivity. In regard to the MSP1M210-18-6W, Figure 4c,d confirm the previously described behavior, with low sensitivity values for voltage under the MPP and, in particular, almost negligible values for the three-measurement setup. In the two-measurement setup the values of sensitivity under the MPP are of order of unit and, again, this is due to the indirect link to the current by means of sensing resistor.

### 3.2. Current Sensitivity and Sensing Resistance Sensitivity

**Figure 5:**
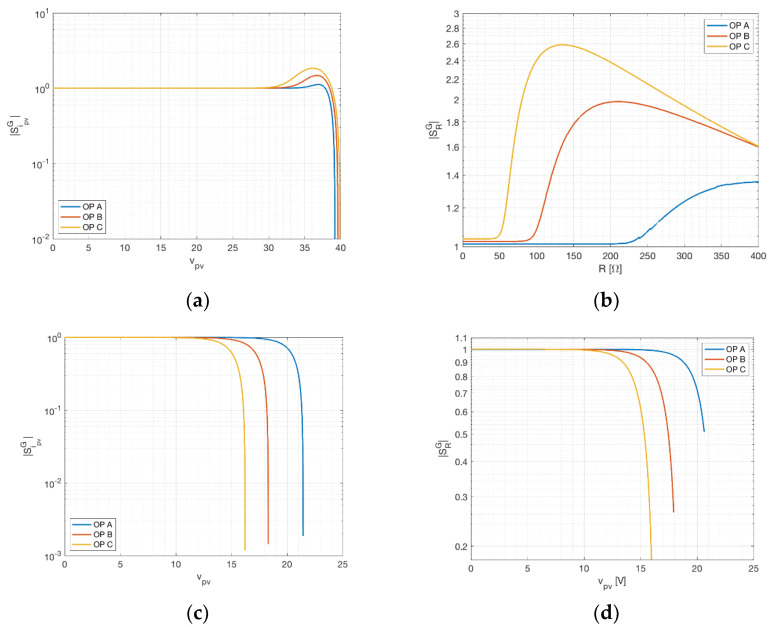
Current (resistance) sensitivity of the irradiance at different operating points (OP). (**a**) The three-measurement approach for STP315S-20. (**b**) The two-measurement approach for KXOB22-01X8L. (**c**) The three-measurement approach and (**d**) the two-measurement one for MSP1M210-18-6W.

Current sensitivity is defined with respect to current in the three-measurement approach by Equation (13) and with respect to sensing resistance in the two-measurement approach by Equation (17). As in the previous case, current sensitivity is shown against panel voltage for different temperatures in Figure 5a. As expected, current sensitivity is very low for operating points near open circuit (since small variations of current happen in this region) and assume a unitary value near short circuit. Concerning the sensitivity of irradiance with respect to sensing resistance, this assumes values between 1 and 3, with a remarkable knee close to the MPP, for KXOB22-01X8L. It is interesting to remark the comparison between $S_{i_{pv}}^{G}$ and $S_{R}^{G}$ for MSP1M210-18-6W, showing an analogous behavior for these two quantities. This might suggest that the role of $S_{i_{pv}}^{G}$ in the three-measurement setup is related to $S_{R}^{G}$ in the two-measurement setup. On the other hand, R is an external quantity which can be fixed during the setup and, consequently, it should have a minimal influence on irradiance evaluation. However, as Figure 5b,c shows, the sensitivity with respect to R assumes values always of the order of 1. Consequently, in this setup, R should be affected by variations as low as possible. Preventive actions should be taken to avoid thermal effects due to overheating and joule dissipation. An additional consequence is the practical infeasibility of using the two-measurement setup for PV modules producing considerable power.

### 3.3. Temperature Sensitivity

**Figure 6:**
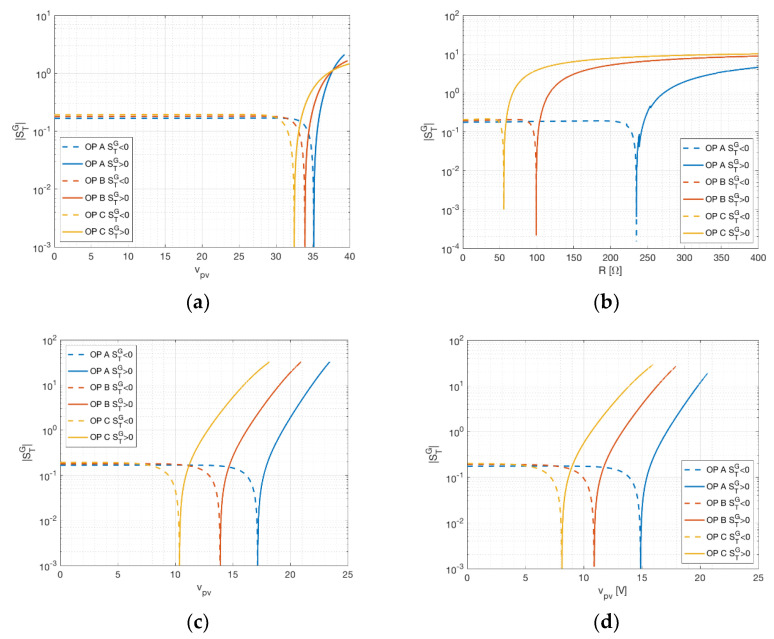
Temperature sensitivity of the irradiance at different operating points (OP). (**a**) The three-measurement approach for STP315S-20. (**b**) The two-measurement approach for KXOB22-01X8L. (**c**) The three-measurement approach and (**d**) the two-measurement one for MSP1M210-18-6W. In this case, (**c**,**d**) are very similar apart from the positions of the minima. Dashed lines represent negative sensitivities, full lines represent positive sensitivities.

Temperature sensitivity is measured in the same way for both the three-measurement and two-measurement approaches and is expressed by Equation (14). As in the previous cases, sensitivity is shown against panel voltage (sensing resistance) for different operating points in Figure 6. As can be seen, in both cases the temperature sensitivity changes sign. This happens for a voltage slightly lower than the voltage at maximum power, identifying a very robust operating condition towards temperature measurement perturbation (clearly in the two-measurement approach, for resistance equivalent to the MPP). The value of the sensitivity is in both case low, before the MPP. This means that error in the measurement of temperature returns lower error in irradiance estimate, at least for operating points lying in the flat region between short circuit and maximum power point. It is worth noticing that for MSP1M210-18-6W the sensitivities with respect temperature, shown in Figure 6c,d, evaluated for the two-measurement setup and for the three-measurement setup, seem practically identical. In fact, they slightly differ in the position of the minima, which, as previous stated, are near the MPP. This difference can be explained by the fact that, in the two-measurement setup, the sensing resistor, which establishes the link between voltage and current, modifies the positions of the minimum of the sensitivity with respect to the temperature, reducing the voltage when this occurs.

### 3.4. Total Sensitivity and Worst-Case Irradiance Perturbation

The individual tests proposed here are useful to determine the optimal operating point, assuming a specific perturbation is expected to be predominant over the others. Although this might be the case, assuming different sensors (with different characteristics) are used for the three quantities, it is seldom the most conservative approach. Observing the sum of the individual sensitivities is the best approach if no prior information on sensor accuracy is known.

The total sensitivity for the three-measurement and two-measurement approaches are given, respectively, by Equations (14) and (15):

(18) $$S_{tot}^{G}=\left|S_{V}^{G}\right|+\left|S_{I}^{G}\right|+\left|S_{T}^{G}\right|\;\;\;\;\;\left(3\;meas.\right)$$

(19) $$S_{tot}^{G}=\left|S_{V}^{G}\right|+\left|S_{R}^{G}\right|+\left|S_{T}^{G}\right|\;\;\;\;\left(2\;meas.\right)$$

**Figure 7:**
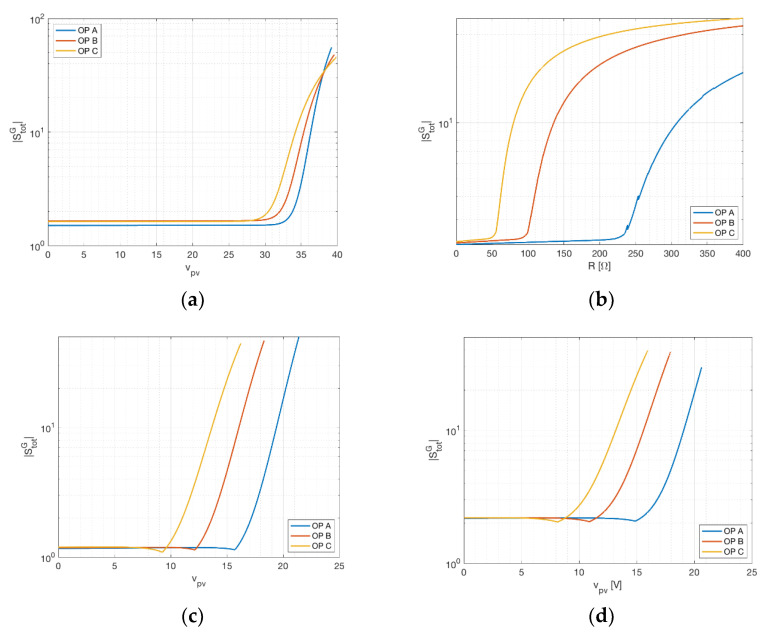
Total sensitivity of the irradiance at different operating points (OP). **(a**) The three-measurement approach for STP315S-20. (**b**) The two-measurement approach for KXOB22-01X8L. (**c**) The three-measurement approach and (**d**) the two-measurement one for MSP1M210-18-6W.

**Figure 8:**
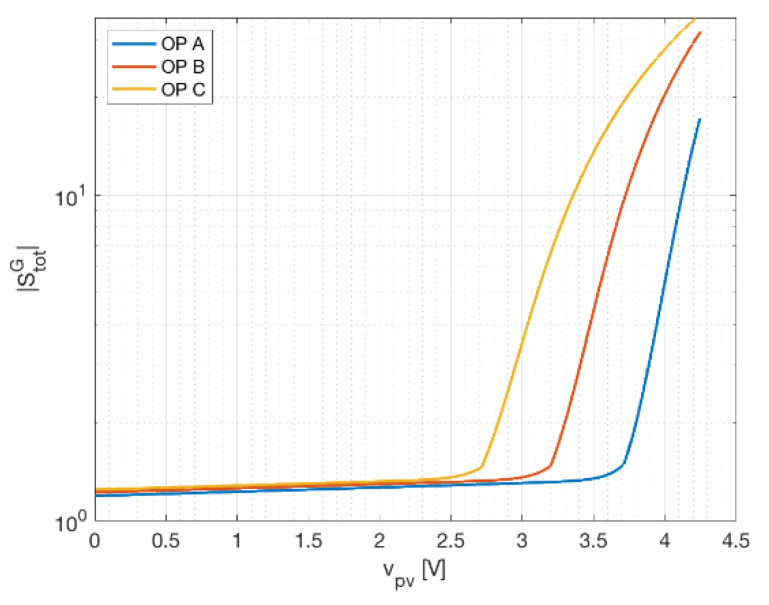
Total sensitivity (vs. voltage) of the irradiance measured with the two-measurement approach (b) for different operating points.

As in the previous cases, sensitivity is shown against panel voltage for different operating points in Figure 7. In order to show that the behavior is really similar, in Figure 8, the total sensitivity for the two-measurement approach is plotted versus voltage. In both cases, voltage lower than the MPP guarantees a total sensitivity lower than 5, resulting in a low amplification of the measurement error. The three-measurement approach underlines that the most conservative approach consists in working with operating voltages, at most, equal to the maximum power point. The two-measurement approach gives good results for sensing resistors lower than the equivalent resistors for the highest MPP admissible (determined considering a value of irradiance between 1200 and 1500 W/m^2^). Lastly, the comparison of the two approaches for MSP1M210-18-6W suggests that the two-measurement approach is more sensitive with respect to the three-measurement one. This is due to the sensing resistor contribution to the total sensitivity. If this contribution is removed, the two cases are practically equal.

Under the assumption that the sensitivities are known, the irradiance perturbation can be derived by little manipulations of Equations (13)–(15). For the worst-case analysis, it is conservative to assume all sensitivities and errors in absolute values. For the three-measurement approach the irradiance perturbation is:

(20) $$\frac{\Delta G}{G}=\left|S_{v_{pv}}^{G}\right|\left|\frac{\Delta V}{V}\right|+\left|S_{i_{pv}}^{G}\right|\left|\frac{\Delta I}{I}\right|+\left|S_{T}^{G}\right|\left|\frac{\Delta T}{T}\right|\;$$

For the two-measurement approach:

(21) $$\frac{\Delta G}{G}=\left|S_{v_{pv}}^{G}\right|\left|\frac{\Delta V}{V}\right|+\left|S_{R}^{G}\right|\left|\frac{\Delta R}{R}\right|+\left|S_{T}^{G}\right|\left|\frac{\Delta T}{T}\right|\;$$

Assuming design choices were made to assure the best working conditions (that is, if the voltage is lower than those at the MPP), $\left|S_{v_{pv}}^{G}\right|\ll \left|S_{i_{pv}}^{G}\right|$ and $\left|S_{T}^{G}\right|<\left|S_{i_{pv}}^{G}\right|$. Thus, the error in the estimation is mainly linked to the error (the tolerance) in the current measurements. This is true for both the methodologies. For the two-measurement approach, in particular, it is safe to assume negligible $\left|\frac{\Delta R}{R}\right|$ (the error on the resistance can be significantly lowered by accurate design). Thus, it is extremely important to measure the voltage accurately. Clearly, the assumption that $\left|\frac{\Delta R}{R}\right|$ is negligible, must be also translated into the fact that the two-measurement setup is not feasible for power PV modules.

### 3.5. Measurement Prototype: The Smart Panel Sensor

A practical instrumental implementation of the three-measurement system is very simple and inexpensive to realize. The instrument should be able to measure independently the operating point of the PV panel and its temperature and be able to derive the power needed for operating directly from the panel itself.

**Figure 9:**
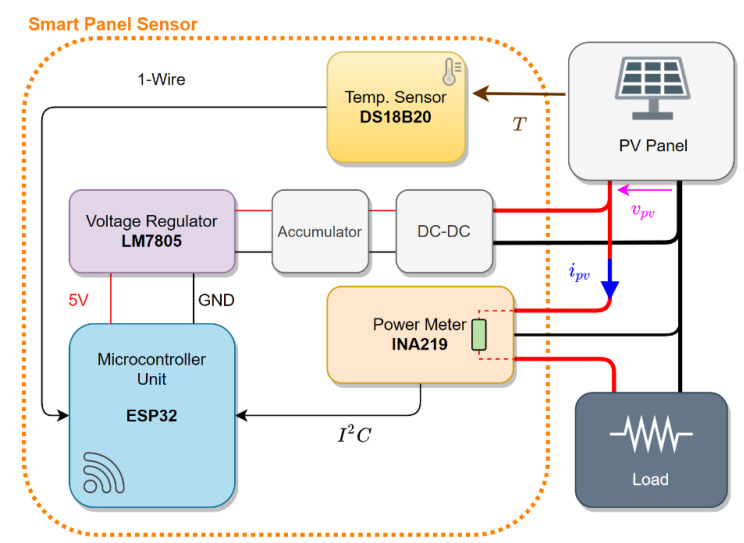
Schematic representation of the smart panel sensor components and connection to a power-producing PV system to achieve the three-measurement approach.

**Figure 10:**
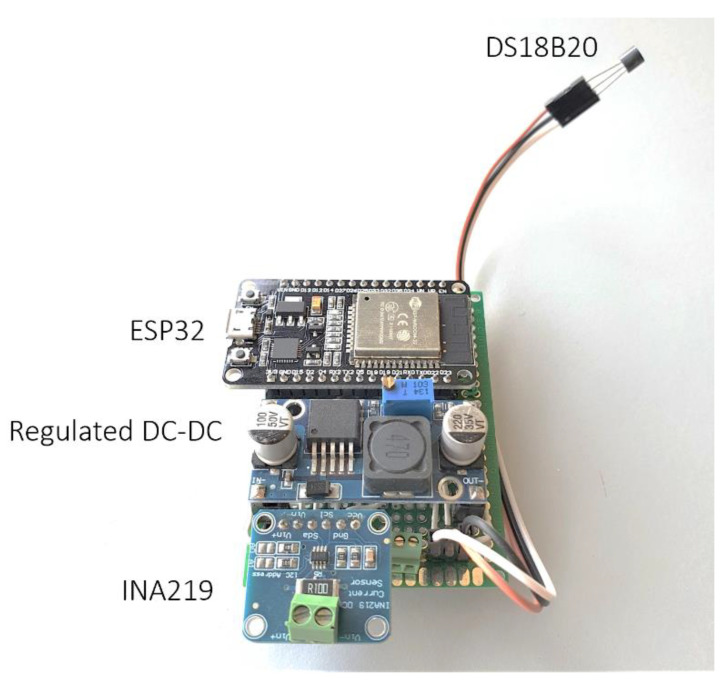
Prototype for the smart panel sensor with main components highlighted.

Moreover, the central processing unit should be equipped with the necessary hardware for wireless communications over Wi-Fi network. A possible implementation is shown in Figure 9. The core of the smart panel sensor is the ESP32 microcontroller by ESPRESSIF. This microcontroller operates with a dual core at 240 MHz, equips a full stack for Wi-Fi 802.11 and Bluetooth 4.2, has several low power modes (and equips a secondary ultra-low power co-processor) and is compatible with most typical communication interfaces. The computing power of the ESP32 can elaborate the raw data from the sensors, compute the irradiance through Equation (8) and transmit data over Wi-Fi without difficulties. Moreover, since the Wi-Fi interface is compatible with mesh topology, the smart panel sensor can be implemented as a node for a wireless sensors network (WSN). Measurement of voltage and current is performed by a dedicated power meter, such as the INA219 by Texas Instruments. This power meter operates on the high side of a source–load configuration and measures both voltage and current on a series resistor, which can be selected to accommodate range and precision of the current measurement. The INA219 communicates with the ESP32 through an I^2^C interface. Temperature measurement can be acquired by a back-side temperature sensor. The DS18B20 temperature sensor by Dallas provides measurements with a resolution up to 12 bits and a typical error curve below 1 K and communicates through the simple 1-Wire protocol. As stated, the power supply should come directly from the PV device. Assuming the PV device is used for power generation, the voltage should be initially scaled by a DC-DC converter. After the converter, a storage (either a battery or a supercapacitor) can be placed for increased reliability. The final adjustment is performed by a dedicated voltage regulator belonging to the LM78xx family. The specific voltage depends on the specific board used for the ESP32. Prototypes running on development boards should use a 5 V regulation and designs mounting the ESP32 directly should use a 3.3 V regulation (since the ESP32 is not 5 V tolerant). A physical implementation of the prototype is shown in Figure 10, where the ESP32, the INA219, the DS18B20 and the regulated DC-DC converter are highlighted.

### 3.6. Prototype Validation Workbench

**Figure 11:**
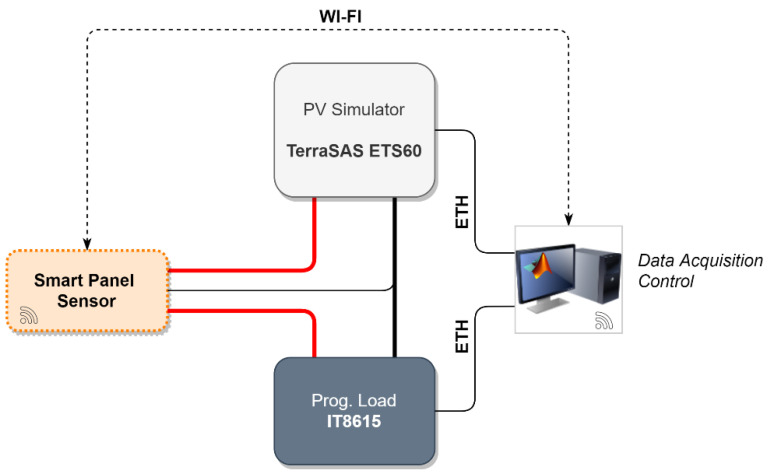
Experimental validation workbench schematic representation.

**Figure 12:**
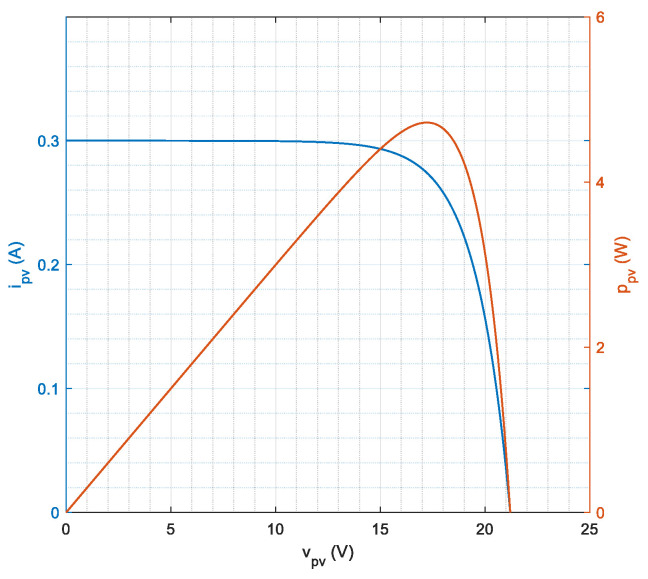
Voltage–current relationship and voltage–power relationship for the simulated PV device used in the experimental validation. The following points define the curve: $V_{OC}=21.20\;V$, $I_{SC}=0.300\;A$, $V_{MP}=17.22\;V$, $I_{MP}=0.274\;A$.

**Figure 13:**
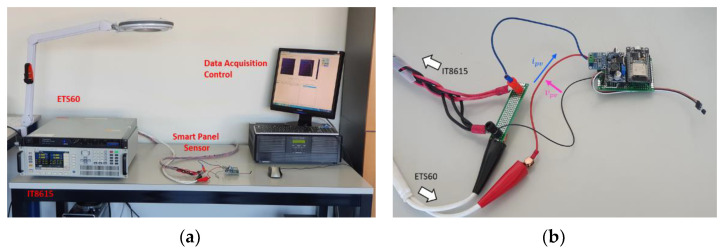
Experimental workbench with highlighted instruments (**a**) and detail on the smart panel sensor connection (**b**).

Validation of the prototype capabilities requires simulating (in a controlled environment) the measurement process that should be performed in the field. A workbench such as the one shown in Figure 11 can be used for this purpose. The photovoltaic source is simulated by means of the TerraSAS ETS60, a 700 W PV simulator. This instrument can reproduce the electrical characteristic of a user-defined PV panel (or string/array) under variable conditions of irradiance and temperature. A programmable DC and AC power load, the IT8615 by Itech, is used to have a realistic power absorption from the PV source. Instrument control is performed via an Ethernet interface in MATLAB r2020 on a dedicated computer. In particular, the irradiance and temperature values for the ETS60 are dynamically changed. Since temperature cannot be measured on a virtual PV device, this information is sent by the data acquisition and control node directly to the smart panel sensor. The smart panel sensor communicates, through Wi-Fi, the recorded irradiance, which is logged and compared against the irradiance set on the ETS60. The ETS60 is programmed to reproduce the electric characteristics shown in Figure 12, which reproduce very closely the MSP1M210-18-6W characteristics. The single-diode model parameters for this device are reported in Table 3. A picture of the experimental workbench, with detail on the smart panel sensor connection, is shown in Figure 13.

**Figure 14:**
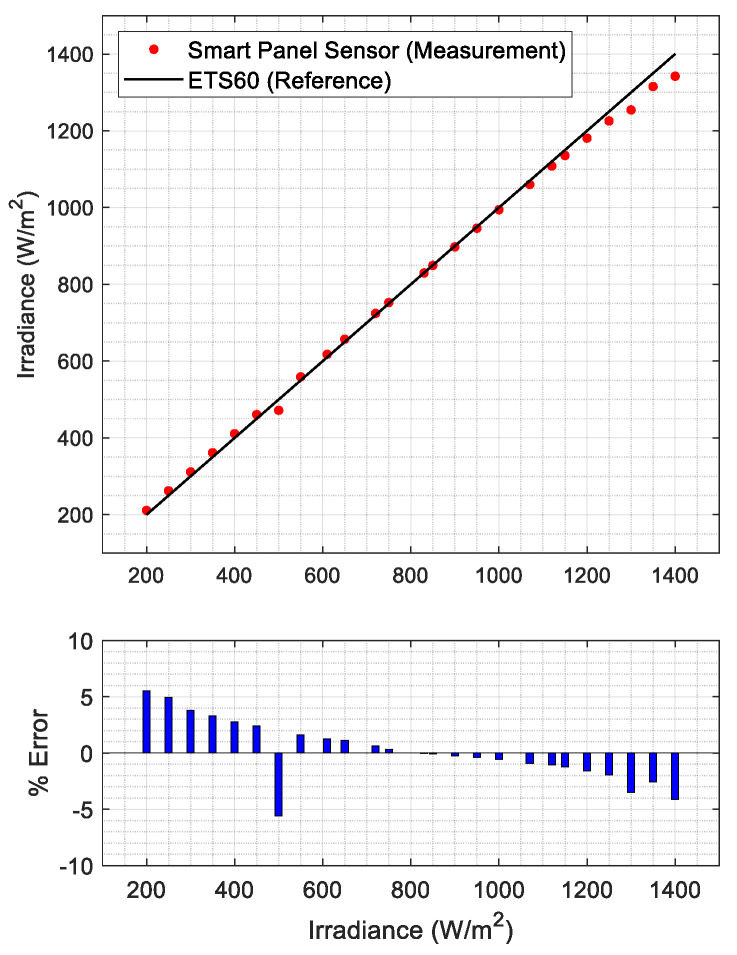
Acquired measurements with the smart panel sensor (**top**) and relative percent error (**bottom**).

The smart panel sensor was tested for a range of irradiances between $200\;W/m^{2}$ and $1400\;W/m^{2}$. As can be seen from Figure 14 (top), the measured irradiances (red dots) lie very closely to the reference irradiance set on the ETS60 (black line). The relative percent error is shown in Figure 14 (bottom). The error is almost always below 5%, which is a very good result, considering the simple nature of the measurement instrument. Moreover, apart from a single outlier at $500\;W/m^{2}$, it clearly exhibits a monotonic, descendent trend. The error can be attributed to several sources, including a low fidelity of the PV simulator to the single-diode model non-SRC operating conditions. However, the regularity of this error trend suggests that, to achieve higher accuracy, a compensation bias could be implemented.

## 4. Discussion

The analysis highlighted different strategies that can be used according to the specific operative scenario of the PV device. Assuming the irradiance is estimated with the three-measurement approach, the device under test will be likely a real PV device connected to a DC-DC converter, driven by an MPPT controller. In this case, an optimal choice of the operating point is impossible, but, as can be seen from Figure 4a and Figure 5a, for operating points near MP the largest sensitivity contribution comes from the current. Focusing on a more accurate current measurement in this case leads to more accurate results in terms of irradiance estimation. However, smaller scenarios such as battery chargers can easily exhibit operating points near short circuit (during charge) and open circuit (during trickle-charge). In the latter case, most of the sensitivity is related to voltage and temperature, as can be seen in Figure 6a. Assuming the application involves the two-measurement approach, the PV device will probably be directly connected to a precision load, possibly with temperature compensation. In this case, the operating point for the PV device can be determined directly by choosing the proper resistance value. As previously stated, the operating area leading to robust measurement lies between the open circuit and MPP, as can be seen in Figure 4b and Figure 5b. Moreover, for voltages slightly lower than the MPP, the minimum value for the temperature sensitivity is found, as can be seen in Figure 6b. Despite the possible advantages concerning a specific operating point, both solutions achieve the desired result of providing an accurate environmental measurement system for a PV device. Prototype implementation showed that the smart panel sensor can be constructed with low-cost devices, increasing the cost of a PV installation only marginally. It should be further stressed that irradiance is, at the same time, the most influential and the most difficult quantity to estimate in PV power production. Direct estimation can be a critical asset for forecasting systems, renewable energy communities and storage management.

## 5. Conclusions

A sensitivity analysis for the closed-form expression of the irradiance on a PV device was discussed in this work, along with a proposed prototype for irradiance measurement in field applications. The approach aimed at giving insight on the robustness of this expression with respect to the measurement setup and the operating point for the device itself. The analysis can be used to quantify uncertainty in several PV applications where irradiance plays an important role, such as MPPT, fault assessment, array reconfiguration, storage management and forecasting.

The prototype described a low-cost solution to transform any PV device in a “smart panel” able to measure the instantaneous incident irradiance and transmit it over wireless network. The prototype was experimentally validated to assess its measurement accuracy.

Two open problems, that will be addressed by future work, arise from the present analysis. The first one involves the comparison of this expression sensitivity versus other approaches based on other methods, such as black-box approaches, or based on different circuit models. The second one involves the statistic investigation of the sensitivity on a large database of devices, such as the CEC database [28], to identify possible trends between the devices constructor parameters and the robust operative points found through sensitivity analysis. The proposed solution, along with the prototype implementation, can be a key asset for smart management of PV power production. This is especially true for the three-measurement approach, which can be implemented easily in already existing systems monitoring the PV operating point for MPPT purposes.

## Figures and Tables

**Table 1:** Circuit parameters for the single-diode models used in the sensitivity analysis and datasheet parameters used for identification.

Model Parameters	STP315S-20	KXOB22-01X8L	MSP1M210-18-6W
$R_{s,ref\;}\left[\Omega \right]$	0.4020	23.896	2.644
$R_{sh,ref\;}\left[\Omega \right]$	186.08	768.27	2686
$I_{irr,ref}\;\left[A\right]$	9.9815	0.0453	0.3
$I_{o,ref}\;\left[A\right]$	8.3563 × 10^−21^	2.6822 × 10^−18^	6.451 × 10^−9^
$n_{ref}$	32.004	4.9136	46.800
Datasheet Parameters			
$V_{OC}\;\left[V\right]$	39.9	5.53	21.2
$I_{SC\;}\left[A\right]$	9.96	0.0059	0.3
$V_{MP}\;\left[V\right]$	33.1	4.46	17.221
$I_{MP}\;\left[A\right]$	9.52	0.0055	0.274
$\alpha_{T\;}\left[\%/K\right]$	0.06	0.48	0.06

**Table 2:** Operating points used for the evaluation of the sensitivity.

Operating Point	Irradiance (W/m^2^)	Temperature (°C)
A	400	15
B	800	45
C	1200	65

**Table 3:** Circuit parameters for the PV simulator validation test.

Parameter	Simulated PV Device
$R_{s,ref\;}\left[\Omega \right]$	2.6441
$R_{sh,ref\;}\left[\Omega \right]$	2686.5
$I_{irr,ref}\;\left[A\right]$	0.3002
$I_{o,ref\;}\left[A\right]$	6.4519 × 10^−9^
$n_{ref}$	46.800

## Appendix A. Quantities and Units of Measurements

In this appendix, symbols used in the paper are summarized in a table along with their description and unity of measurement. For entries without a unity of measurement, the symbol represents a pure number.

**Table A1:** Symbols and unity of measurements.

Symbol	Description	Unity of Measurement
$i_{pv}$	PV device current	(A)
$v_{pv}$	PV device voltage	(V)
$p_{pv}$	PV device power (active sign convention)	(W)
$I_{irr}$	Photogenerated Current	(A)
$R_{S}$	Series Resistance	(Ohm)
$R_{SH}$	Shunt Resistance	(Ohm)
$I_{O}$	Reverse saturation current	(A)
n	Ideality Factor	
k	Boltzmann Constant	(eV/K)
$E_{g}$	Silicon Bandgap Energy	(eV)
T	Cell temperature	(K)
G	Solar irradiance	(W/m^2^)
$V_{OC}$	PV device open circuit voltage	(V)
$I_{SC}$	PV device short circuit current	(A)
$V_{MP}$	PV device maximum power voltage	(V)
$I_{MP}$	PV device maximum power current	(A)
$\alpha_{T}$	PV device short circuit current temperature coefficient	(A/K)
$S_{X}^{Y}$	Sensitivity of Y with respect to X	

## References

[1] Diagne M., David M., Lauret P., Boland J., Schmutz N. (2013). Review of solar irradiance forecasting methods and a proposition for small-scale insular grids. Renew. Sustain. Energy Rev..

[2] Chaibi Y., Allouhi A., Malvoni M., Salhi M., Saadani R. (2019). Solar irradiance and temperature influence on the photovoltaic cell equivalent-circuit models. Sol. Energy.

[3] De Soto W., Klein S., Beckman W. (2006). Improvement and validation of a model for photovoltaic array performance. Sol. Energy.

[4] King D.L. (1997). Photovoltaic module and array performance characterization methods for all system operating conditions. AIP Conf. Proc..

[5] Du Y., Fell C., Duck B., Chen D., Liffman K., Zhang Y., Gu M., Zhu Y. (2016). Evaluation of photovoltaic panel temperature in realistic scenarios. Energy Convers. Manag..

[6] Udenze P., Hu Y., Wen H., Ye X., Ni K. (2018). A Reconfiguration Method for Extracting Maximum Power from Non-Uniform Aging Solar Panels. Energies.

[7] Ali M., Mahmoud K., Lehtonen M., Darwish M. (2021). Promising MPPT Methods Combining Metaheuristic, Fuzzy-Logic and ANN Techniques for Grid-Connected Photovoltaic. Sensors.

[8] Scolari E., Sossan F., Paolone M. (2017). Photovoltaic-Model-Based Solar Irradiance Estimators: Performance Comparison and Application to Maximum Power Forecasting. IEEE Trans. Sustain. Energy.

[9] Laudani A., Lozito G.M. (2019). Smart Distributed Sensing for Photovoltaic Applications. 2019 PhotonIcs & Electromagnetics Research Symposium—Spring (PIERS-Spring).

[10] Kumar D.S., Yagli G.M., Kashyap M., Srinivasan D. (2020). Solar irradiance resource and forecasting: A comprehensive review. IET Renew. Power Gener..

[11] Brenna M., Foiadelli F., Longo M., Zaninelli D. (2018). Energy Storage Control for Dispatching Photovoltaic Power. IEEE Trans. Smart Grid.

[12] Pei T., Hao X. (2019). A Fault Detection Method for Photovoltaic Systems Based on Voltage and Current Observation and Evaluation. Energies.

[13] Oliveri A., Cassottana L., Laudani A., Fulginei F.R., Lozito G.M., Salvini A., Storace M. (2015). Two FPGA-Oriented High-Speed Irradiance Virtual Sensors for Photovoltaic Plants. IEEE Trans. Ind. Inform..

[14] Carrasco M., Laudani A., Lozito G.M., Mancilla-David F., Fulginei F.R., Salvini A. (2017). Low-Cost Solar Irradiance Sensing for PV Systems. Energies.

[15] Moreno-Garcia I.M., Palacios-Garcia E.J., Pallares-Lopez V., Santiago I., Redondo M.J.G., Varo-Martinez M., Calvo R.J.R. (2016). Real-Time Monitoring System for a Utility-Scale Photovoltaic Power Plant. Sensors.

[16] Madeti S.R., Singh S. (2017). A comprehensive study on different types of faults and detection techniques for solar photovoltaic system. Sol. Energy.

[17] Madeti S.R., Singh S. (2017). Online fault detection and the economic analysis of grid-connected photovoltaic systems. Energy.

[18] Eghtedarpour N., Farjah E. (2012). Control strategy for distributed integration of photovoltaic and energy storage systems in DC micro-grids. Renew. Energy.

[19] Zaouche F., Rekioua D., Gaubert J.-P., Mokrani Z. (2017). Supervision and control strategy for photovoltaic generators with battery storage. Int. J. Hydrogen Energy.

[20] Marcos J., De La Parra I., García M., Marroyo L. (2014). Control Strategies to Smooth Short-Term Power Fluctuations in Large Photovoltaic Plants Using Battery Storage Systems. Energies.

[21] Gilman P., Diorio N.A., Freeman J.M., Janzou S., Dobos A., Ryberg D. (2018). SAM Photovoltaic Model Technical Reference Update.

[22] Toledo F., Blanes J.M., Galiano V., Laudani A. (2021). In-depth analysis of single-diode model parameters from manufacturer’s datasheet. Renew. Energy.

[23] Coco S., Laudani A., Lozito G.M., Fulginei F.R., Salvini A. (2019). Sensitivity analysis of the reduced forms of the one-diode model for photovoltaic devices. Int. J. Numer. Model. Electron. Netw. Devices Fields.

[24] Toledo F.J., Blanes J.M., Galiano V. (2018). Two-Step Linear Least-Squares Method For Photovoltaic Single-Diode Model Parameters Extraction. IEEE Trans. Ind. Electron..

[25] Wiyadi E., Wati A., Hamzah Y., Umar L. (2020). Simple I-V acquisition module with high side current sensing principle for real time photovoltaic measurement. J. Phys. Conf. Ser..

[26] Ngo G.C., Floriza J.K.I., Creayla C.M.C., Garcia F.C.C., Macabebe E.Q.B. Real-time energy monitoring system for grid-tied Photovoltaic installations. Proceedings of the TENCON 2015—2015 IEEE Region 10 Conference.

[27] Laudani A., Fulginei F.R., Salvini A. (2014). Identification of the one-diode model for photovoltaic modules from datasheet values. Sol. Energy.

[28] Dobos A.P. (2012). An Improved Coefficient Calculator for the California Energy Commission 6 Parameter Photovoltaic Module Model. J. Sol. Energy Eng..

